# A Dieting Facilitator on the Fridge Door: Can Dieters Deliberately Apply Environmental Dieting Cues to Lose Weight?

**DOI:** 10.3389/fpsyg.2020.582369

**Published:** 2020-12-21

**Authors:** Aline E. Stämpfli, Sabrina Stöckli, Thomas A. Brunner, Claude Messner

**Affiliations:** ^1^Department of Consumer Behavior, Institute of Marketing and Management, University of Bern, Bern, Switzerland; ^2^Food Science and Management, School of Agricultural, Forest, and Food Sciences HAFL, Bern University of Applied Sciences, Zollikofen, Switzerland

**Keywords:** dieting, environmental cue, awareness, restrained eating, longitudinal study

## Abstract

Individuals exposed to dieting-related environmental cues have been repeatedly shown to be better able to resist tempting food. This especially applies to restrained eaters who hold a chronic dieting goal. Thus far, mainly short-term effects of environmental dieting cues have been examined and the individuals were typically unaware of being influenced. Yet, it is unclear whether individuals can deliberately apply environmental dieting cues for themselves to facilitate the pursuit of the longer-term goal of losing weight. The present longitudinal study applied a 2 (cue: visually dieting-related vs. visually neutral cue) × 2 (awareness: being aware vs. not being aware of the cue’s facilitating influence) between-subjects design for 6 months (*N* = 166 participants who started the study; *M*_*age*_ = 47.85 years; 69.9% female; *M*_*BMI*_ = 29.07 kg/m^2^). Our results provide preliminary indications that cue, awareness, and restrained eating interact. The results suggest that high (vs. low) restrained eaters could deliberately apply environmental dieting cues for themselves to facilitate losing weight. However, further studies are needed to explore the effects of environmental dieting cues over a longer period of time.

## Introduction

Our *obesogenic* environment, with its abundance of tempting food and food-related cues (Papies et al., 2014; Enax et al., 2015; Papies, 2016b, 2017; Watson et al., 2017), is one of the predominant explanations for the global obesity epidemic (Keith et al., 2006; McAllister et al., 2009; World Health Organization [WHO], 2018, 2020b).

Fortunately, for individuals with a dieting goal, cues in the environment related to dieting can have an influence in the opposite direction: individuals exposed to environmental dieting cues have been shown to eat both less and healthier. For example, a screensaver showing a sculpture by Alberto Giacometti, with humans of exaggeratedly thin proportions, led individuals to eat less in a chips tasting (Stämpfli and Brunner, 2016). Similarly, when a poster depicting a Giacometti sculpture was placed near a snack vending machine, consumers were more likely to choose healthy over unhealthy snacks than when either no poster or a poster showing the hedonic motive of a fun fair was placed nearby (Stöckli et al., 2016).

These examples indicate that individuals were mostly exposed to dieting cues by others, without being aware that they were being influenced. In addition, mainly the short-term effects of environmental dieting cues were examined, such as the vending machine poster’s immediate effect on consumers’ snack choices (Stöckli et al., 2016; see also Buckland et al., 2018). Yet, there remains a research gap regarding whether individuals can deliberately apply environmental dieting cues for themselves to facilitate the pursuit of their longer-term goal of losing weight. The present study has addressed this research gap.

### Awareness Could Hinder or Facilitate Environmental Cues’ Effects

Whether individuals are aware that they are influenced by an environmental cue or not could be decisive for the cue’s effects. On the one hand, individuals who are aware that they are being influenced may attempt to correct for the influence (Wegener and Petty, 1995; see Mussweiler and Damisch, 2008). According to psychological reactance theory, this is because individuals try to restore their freedom of choice when they feel it to be threatened (Brehm, 1966; Miron and Brehm, 2006). Attempting to restore one’s freedom of choice can result in what has been termed “contrast effects”—that is, diminished or reversed effects of the threatening external stimuli such as environmental cues (e.g., Lombardi et al., 1987; Strack et al., 1993; see further Zhu et al., 2015). Individuals’ freedom of choice can even be threatened by internal self-imposed threats, arising from choosing specific alternatives and having to give up on others (Iyengar and Lepper, 2000; Miron and Brehm, 2006; Steindl et al., 2015). Thus, even self-imposed dieting-compatible behaviors could evoke reactance over time (Ungar et al., 2015).

However, there is evidence that contrast effects do not occur even though individuals are aware of being influenced. For example, when healthy instead of unhealthy snacks were placed near the cash register at train station snack shops, customers more often chose healthy snacks. This effect was maintained when a sign that read “We help you make healthier choices” was placed near the cash register (Kroese et al., 2016; see further Payne et al., 2016; Bruns et al., 2018). Moreover, individuals have been shown to successfully use environmental cues as reminders to implement their intentions. For instance, coffee shop customers who accepted a $1 coupon toward a future purchase along with a flyer that depicted a stuffed alien and mentioned the alien would be on the cash register as a reminder to redeem the coupon were more likely to use the coupon 2 days later when a stuffed alien was placed at the cash register, compared with customers who received a flyer that did not announce the stuffed alien (Rogers and Milkman, 2016).

### Environmental Cues Drive Behavior by Activating Highly Valued Goals

While the role of awareness regarding the effects of environmental cues has as yet not been clearly determined, previous research has established the important role of goals (see Papies, 2017). The prominently discussed mechanism of how environmental cues influence behavior is that of activating goals (Förster et al., 2007; Papies et al., 2008; Papies, 2016a,b, 2017; Weingarten et al., 2016). This is possible as goals are embedded in cognitive structures that interlink the goals with mental representations of distinct environmental cues and behaviors. Such cognitive structures emerge when individuals repeatedly perform a behavior with similar goals in the same context (Papies, 2016b). For example, an individual’s repeated dieting attempts with the goal of losing weight may be linked to diet products; subsequently, seeing such diet products can activate the individual’s dieting goal (Anschutz et al., 2008b).

Goals are assumed to drive goal pursuit as they represent end states associated with a reward or positive affect (see Custers and Aarts, 2010). Correspondingly, activated goals have been shown to have stronger effects on behavior when the positive affect related to the goals was higher—in other words, when goals were highly valued by the individuals holding the goals (see Bargh, 2016; see Papies, 2016b; Weingarten et al., 2016). Also in line with this, environmental dieting cues have been shown to have a particularly pronounced effect on individuals who hold a chronic dieting goal, the restrained eaters (Herman and Mack, 1975; Herman and Polivy, 1980; Weingarten et al., 2016; Polivy and Herman, 2017; see Buckland et al., 2018; Masterson et al., 2019). For example, only restrained eaters tasted fewer meat samples in a butcher shop when exposed to a poster on the entrance door that announced a weekly recipe as being low-calorie and “good for a slim figure” (Papies and Hamstra, 2010). Similarly, the portrayal of the exaggeratedly thin Giacometti figures (Stämpfli and Brunner, 2016) was found to specifically influence restrained eaters (Stämpfli et al., 2017). A meta-analysis of the effects of environmental dieting cues on food intake supported the important role of dieting goals. Overall, dieting cues were found to reduce food intake only to a minor extent. In contrast, for individuals with a strong dieting goal, including both restrained eaters and dieters, a small-to-moderate effect was found (Buckland et al., 2018). While restrained eaters are defined as having a chronic dieting goal or “concern for dieting” (see section “Measures”; Dinkel et al., 2005), dieters can be characterized as actively engaging in a weight management attempt at the moment and thus having a current dieting goal.

Overall, based on the presented research, we expected that individuals with restrained eating tendencies, and thus a chronic dieting goal per definition, might be able to successfully apply environmental dieting cues for themselves to facilitate the pursuit of their longer-term goal of losing weight.

### Scant Evidence on Environmental Dieting Cues’ Effects Over a Longer Period of Time

In addition to the question of whether individuals can deliberately apply environmental cues for themselves to facilitate the pursuit of a personal goal, the present study addressed a second question that, to date, has received little attention: whether or not environmental cues in general (van Kleef and van Trijp, 2018) and dieting cues in particular can be applied effectively over a longer period of time (Papies, 2016b; Buckland et al., 2018). This is essential regarding the dieting goal, as losing weight requires time.

The scant evidence on environmental dieting cues which are applied over a longer period of time has revealed ambiguous findings. On the one hand, two studies on snacking frequency over 2 weeks indicate that an environmental dieting cue (a photograph of a slim model) can reduce the influence of unhealthy eating habits (Ohtomo, 2017). On the other hand, a similar environmental dieting cue (a picture of a slim model) was found not to help individuals lose weight during two “weight loss programs” that utilized eating diaries over periods of 1 week (Klesse et al., 2012; see for a further example, Stice et al., 2001). In the first weight loss program, the cover of an eating diary depicted either a thin model or a measuring tape; in the second, the cover of the eating diary depicted either a thin model or the same model adjusted to appear normal in size. At the end of a week, the participants exposed to a thin model had gained some weight or lost less weight compared with the participants exposed to the normal-size model or the measuring tape. The results of these studies were explained by the discouraging effect of the thin models which made the participants perceive their weight loss goals as being less attainable (Klesse et al., 2012).

## Overview of the Present Research

The present study explored the influence of environmental dieting cues on self-declared dieters over 6 months to address the research gap on the effects of environmental dieting cues over a longer period of time (Buckland et al., 2018). Moreover, it examined whether environmental dieting cues can be applied in a self-directed and deliberate manner by individuals to facilitate the pursuit of a personal goal. A 2 (cue: visually dieting-related vs. visually neutral cue) × 2 (awareness: being aware vs. not being aware of the cue’s facilitating influence) between-subjects design was applied, with weight loss as the dependent variable.

In the two experimental conditions in which individuals were intended to be aware of the cue’s (supposed) effect, the cues were presented to them as dieting facilitators. Thus, the cues could be assumed to be in line with the dieters’ goal to lose weight and with restrained eaters’ chronic dieting goal (Polivy and Herman, 2017). We therefore expected to find no contrast effects (see Rogers and Milkman, 2016). However, we did expect that deliberately applying the (supposed) dieting cues would help the participants in the experimental awareness conditions to lose weight as compared with participants assigned to the control condition, in which individuals were given a visually neutral cue that was not presented to them as a dieting facilitator. Our assumption that the visually neutral cue could facilitate weight loss when it was presented as being a dieting facilitator was based on previous evidence that cues visually unrelated to a goal that are established as a reminder of the goal can still support goal pursuit (Rogers and Milkman, 2016). Finally, regarding the experimental condition in which participants applied an environmental cue that was not presented to be a dieting facilitator but that was visually related to dieting, we hypothesized that the cue would help the participants to lose weight compared with participants in the control condition, consistent with the main body of evidence that individuals are influenced by environmental cues without being aware of them (Chartrand, 2005; see Buckland et al., 2018).

## Methods

### Design

A 2 (cue: visually dieting-related vs. visually neutral cue) × 2 (awareness: being aware vs. not being aware of the cue’s facilitating influence) between-subjects design was applied, with losing weight over time as the dependent variable. This design resulted in a condition in which participants applied the visually neutral cue which was not presented to them as being a dieting facilitator, the control condition *Rothko not aware* (see section “Cues”), and three experimental conditions related to dieting: one in which participants applied a visually dieting-related cue which was not presented to them as being a dieting facilitator, the experimental condition *Giacometti not aware*, and two in which participants were told that their cue had been shown to facilitate dieting in the past and in which the participants either applied a visually neutral cue (experimental condition *Rothko aware*) or a visually dieting-related cue (experimental condition *Giacometti aware*).

### Materials and Measures

#### Cues

The visually dieting-related cue consisted of pictures of sculptures by the artist Alberto Giacometti (Brunner and Siegrist, 2012). These sculptures depict humans of exaggeratedly thin proportions. The primary cue was a picture of the sculpture *Piazza*. In addition, a picture of the sculpture *L’homme qui marche I* was applied. The visually neutral cue was a picture of the Mark Rothko painting *No. 203 c.1954*, which depicts a blue and a pinkish field. They can be viewed by conducting Google image searches using the keywords “Giacometti Piazza,” “Giacometti l’homme qui marche I,” and “Rothko 203,” respectively. The cues were printed on and in dieting diaries (DIN A5-sized) in which the study participants were asked to record their weight daily as well as their physical activity and their fruit and vegetable consumption. In addition, each participant received six stickers (10.50 × 7.40 cm/4.13 × 2.91″) that depicted their cue.

Approximately half of each diary’s front cover displayed either the primary visually dieting-related cue (Giacometti’s *Piazza*) or the visually neutral cue (the Rothko painting). The supporting visually dieting-related cue (Giacometti’s *l’homme qui marche I*) or the visually neutral cue were printed in a smaller size (roughly 1/8 of the page) on each page of the diary, including on the diary’s spine. Furthermore, each of the pages included either the primary visually dieting-related cue (Giacometti’s *Piazza*) or the visually neutral cue as the background of the table in which participants recorded their weight, occupying roughly 1/4 of the page.

#### Awareness

Study participants in the two experimental “awareness conditions” were told that the visually dieting-related pictures of the Giacometti sculptures or the picture of the visually neutral Rothko painting, respectively, were intended to serve as reminders of their participation in the study and, importantly, that the pictures were dieting facilitators. More precisely, participants were told that the cues had been proven to support dieting in the past by making people eat less and healthier. This was communicated orally at the study briefing as well as in writing on the introduction page of the dieting diaries. In the “no-awareness conditions,” participants were not told that the images on their diaries and stickers were dieting facilitators. They were only told that the stickers were intended as reminders of their participation in the study. All participants were encouraged to place the stickers where they would most likely influence their eating behaviors, such as posted on the fridge door, on a mirror, or in the dining room.

#### Measures

Participants’ percentage changes in body weight, as derived from weighing the participants at the briefing and at the debriefing of the study using a body composition monitor scale, were used as the dependent variable.

Since restrained eaters are especially sensitive to dieting-related environmental cues (Papies and Hamstra, 2010; Stämpfli et al., 2017), restrained eating was captured at the briefing using the German version (Dinkel et al., 2005) of the concern for dieting subscale (α = 0.66) of the Revised Restraint Scale (Herman and Polivy, 1980). The use of the concern for dieting subscale has been shown to capture restrained eating better than the use of the entire restraint scale (van Strien et al., 2002). The subscale consists of six items, which were reformulated in the first person, and were assessed on a 7-point Likert scale (1 = “I do not agree at all” to 7 = “I entirely agree”). Example items are: “I often diet,” “I give too much time and thought to food,” and “I have feelings of guilt after overeating.” It is important to note that restrained eating assessed by the Revised Restraint Scale has been shown (Laessle et al., 1989; see Stroebe et al., 2013) to be related to disinhibited eating or less successful dieting, compared with restrained eating assessed by other measures, such as the Three-Factor Eating Questionnaire (TFEQ; Stunkard and Messick, 1985) and the Dutch Eating Behavior Questionnaire (DEBQ; van Strien et al., 1986).

At the end of the study, participants were asked whether they had used the stickers on a 7-point Likert scale (1 = “I do not agree at all” to 7 = “I entirely agree”) and approximately how often a day they had seen the stickers (“less than once,” “once,” “twice,” “three times,” “four times,” and “almost always”). In addition, participants were asked where they had affixed the stickers and were given the option to select answers and/or to provide their own. Finally, participants were asked an open question about what they thought was the study’s purpose.

### Participants

#### Recruitment Process

To recruit study participants, approximately 20,000 flyers were dispatched by post in and around the suburban location of the university where the study was conducted and where the study’s briefing and debriefing took place. The flyers asked “Are you still on your way to your ideal weight?” and sought to reach individuals with pre-established weight loss goals who were interested in taking part in a 6-month dieting study using dieting diaries. The flyer was also posted inside an organization in the place where the university is located, and members of the university’s consumer panel were invited to participate via e-mail. The call for study participants was also published on the internet (e.g., the university’s homepage) and in free newspapers.

As an incentive for participation, participants were told they would receive edited reports on the self-recorded individual data from the study, after the study had ended, showing their personal weight progressions during the study in figures as well as the data from a body composition monitor scale that was used during the study’s briefing and debriefing sessions (e.g., body fat and muscle mass). These personal reports showed comparisons between the individual’s values at the beginning and end of the study.

Interested individuals could register for the study online. Only those with a body mass index (BMI; computed as a person’s weight divided by the square of the person’s height, kg/m^2^) of >22 were admitted, resulting in the exclusion of 12 individuals. This BMI limit was chosen to ensure that the study would not encourage weight loss below the BMI range of normal weight, which is 18.5–24.9 (World Health Organization [WHO], 2020a). A BMI of 21.7 is the middle point of this BMI range, and our lower limit of 22 thus allowed weight loss within the normal weight range. Further, no participant was accepted who was less than 20 years of age.

#### Initial Briefing

Admitted participants were asked to register online for a 1-hour appointment for an initial briefing. Appointments were offered at the university from 9:00 a.m. to 8:00 p.m. (except from 12 noon to 1:00 p.m.) during a 2-week period. A maximum of five participants could register per appointment. The control condition and the three experimental conditions (see section “Design”) were then assigned to the appointments with the goal of counterbalancing gender and BMI, as well as the time of day of the initial appointment, among the conditions. In seven cases, two individuals from one household participated in the study. In these cases, both individuals from the household were assigned to the same condition, and the households with two participants were distributed among the conditions.

In total, 167 individuals participated in the study briefing at the university. One of them unsubscribed during the briefing because of a heart pacemaker which did not allow this participant to be weighed using the body composition monitor scale. One hundred sixty-six participants started the study (*M*_*age*_ = 47.85 years, *SD*_*age*_ = 14.62; *M*_*BMI*_ = 29.07 kg/m^2^, *SD*_*BMI*_ = 4.27 kg/m^2^; 69.9%, *n* = 116 female). With 29.07 kg/m^2^, the participants’ mean BMI at the beginning of the study was at the upper limit of the overweight range, which is 25.0 to 29.9 kg/m^2^ (World Health Organization [WHO], 2020a). Sixty-one participants had a BMI in the obesity range (≥30 kg/m^2^). The mean BMIs did not differ by condition, *F*(3,162) = 1.45, *p* = 0.231.

#### Participant Dropouts

Of the 166 participants who started the study, 54 did not attend the weighing at the debriefing of the study and dropped out. Although participants were not required to give a reason for quitting the study, some mentioned dropping out due to illness, illness of a partner, stress in daily life, having an irregular daily life, being stressed by the study, lack of motivation, little endurance, not feeling able to successfully pursue their weight loss goal, having achieved their weight loss goal, or having become satisfied with their weight. The data of the participants who dropped out of the study were not included in the analyses. In addition, the data of five participants who attended the debriefing but had noted their weight in the dieting diary too unreliably and irregularly were not included in the analyses, as it was uncertain whether these participants seriously participated in the study. The self-reported weight data of participants who participated in the study until the end but did not attend the weighing at the debriefing were not analyzed.

A dropout analysis found that participants who dropped out or whose data were not considered for the analyses were equally distributed among the conditions [χ^2^(3,167) = 3.11, *p* = 0.375]. The numbers of participants per condition, compared at the beginning and at the end of the study, were as follows: control condition *Rothko not aware* (i.e., visually neutral cue, not presented as a dieting facilitator, *n* = 42/24), experimental condition *Giacometti not aware* (i.e., visually dieting-related cue, not presented as a dieting facilitator, *n* = 41/30), experimental condition *Rothko aware* (i.e., visually neutral cue, presented as a dieting facilitator, *n* = 41/24), and experimental condition *Giacometti aware* (i.e., visually dieting-related cue, presented as a dieting facilitator, *n* = 43/29).

#### Remaining Study Sample

The data of 107 participants (*M*_*age*_ = 48.73 years, *SD*_*age*_ = 13.69; *M*_*BMI*_ = 28.28 kg/m^2^, *SD*_*BMI*_ = 4.08 kg/m^2^; 67.3%, *n* = 72 female) remained for the analyses. One participant who applied the visually dieting-related Giacometti cue which was not presented as being a dieting facilitator (see section “Design”) stated that she or he thought that the purpose of the study was to find out whether participants lost more weight when they “had the pictures than when they did not have the pictures.” The data of this participant were not excluded from the analyses as it was not entirely clear whether the statement referred to the stickers in general (see section “Cues”) or the motif (the Giacometti picture). Either way, the results of the analyses with *N* = 106 participants did not deviate from the results of the analyses with *N* = 107 participants. The data of participants who reported (via contacting the study team, making notes in their dieting diaries, or providing information during their debriefing appointments) circumstances that could have influenced their weight loss—such as holidays, illness, or concurrent dieting support from a physician—were likewise not excluded from the analyses. Since individuals trying to lose weight in daily life are likely to confront both mitigating and supporting influences such as these, the data of the respective participants were included to support the study’s ecological validity.

### Procedure

Briefings for the study were held at the university campus in November during the 2 weeks before the beginning of the study. The briefing sessions were held separately for each condition, with a maximum of five participants in each briefing session.

At the briefing, the participants were first asked to sign a written informed consent form which explained that they would be weighed with a body composition monitor scale at the beginning and end of the study and that they would have to record their weight daily during the 26 weeks of the dieting study. They were also informed that the study team would not include any physicians or nutrition counselors and that it would be the participants’ responsibility to evaluate if it would be advisable to contact a physician due to their participation in the study. They were advised that they should only participate if they felt psychologically and physically able to do so and if there were no medical reasons for them not to participate. In addition, participants were informed that they had the right to end their participation at any time without giving a reason and that weight loss below the normal weight range was not encouraged. Finally, they were asked not to discuss the study with other participants.

After the participants gave their written informed consent, also at the briefing, they answered the restrained eating questions, received their dieting diaries and adhesive stickers, and were verbally instructed on how to use the stickers and how to record their data. Depending on the condition, they were either informed or not that their cues were dieting facilitators.

The participants were then weighed individually on the body composition monitor scale (Tanita BC-545). They removed shoes and heavy clothing items when they were weighed. In addition, care was taken that participants removed jewelry and belts and had no electronic medical implants in order to ensure valid and safe measurements. In an attempt to prevent any encouragement of unhealthy diets, all participants received a flyer that stated that it should not be their goal to lose weight as fast as possible, but instead to develop a joy in eating in a healthy and well-balanced manner over the long run. To support participants’ motivation to participate in the study, the flyer also stated that weight fluctuations are a normal part of dieting.

The study began in the first week of December and ran for 26 weeks, until the end of May. In June, participants attended debriefing sessions, which took place in the same location as where the briefings had been held. Most of the participants (88.8% of the 107 remaining in the study sample) were weighed in the first week after the study had ended, and some of them were weighed in the second week (5.6%) or third week after the study’s end (more precisely after 17 days, also 5.6%). At the debriefing, the dieting diaries were collected, and participants were measured individually using the body composition monitor scale. Afterwards, all participants were informed of the study’s purpose and were debriefed, both verbally and in writing.

## Results

### Exposure to the Cues

The data of 107 participants were analyzed. Regarding the exposure to the cues, it is important to note that all participants were exposed to the cues in their dieting diaries in which they had been advised to record their weight daily. Regarding the use of the stickers that depicted the cues, 54.2% of the 107 participants stated that they applied the stickers (indicating a value of 5 or higher on a 7-point scale), and 40.2% stated that they had not applied the stickers (indicating a value of 3 or lower). The first group saw the stickers about four times per day on average according to their own statements. An analysis of variance (ANOVA) with cue, awareness, and their interaction revealed that the sticker usage did differ depending on the participants being in an awareness condition or not, *F*(1,103) = 4.07, *p* = 0.046, η^2^_*p*_ = 0.04 (adjusted *R*^2^ = 0.032). Participants to whom the cues were presented as dieting facilitators (i.e., participants in the awareness conditions) agreed more strongly that they used the stickers (*M* = 4.72, *SD* = 2.17) than participants to whom the cues were not presented as dieting facilitators (i.e., participants in the no awareness conditions, *M* = 3.76, *SD* = 2.46). There was no main effect of cue, that is, participants who applied the visually dieting-related cue did not agree more strongly that they used the stickers than participants who applied the visually neutral cue, *F*(1,103) = 1.09, *p* = 0.299, η^2^_*p*_ = 0.01, or an interaction of cue and awareness, *F*(1,103) = 0.83, *p* = 0.364, η^2^_*p*_ = 0.01. The participants (*N* = 107) most often affixed the stickers in their kitchen (most commonly on or in the fridge), on items that they brought with them “on the go,” and in the eating area.

### Three-Way Interaction of Cue, Awareness, and Restrained Eating

An ANOVA with cue, awareness, and their interaction revealed no main effect of cue, *F*(1,103) = 0.29, *p* = 0.594, η^2^_*p*_ = 0.00, or awareness, *F*(1,103) = 2.28, *p* = 0.135, η^2^_*p*_ = 0.02, on participants’ percentage weight change. Cue and awareness also did not interact, *F*(1,103) = 0.15, *p* = 0.703, η^2^_*p*_ = 0.00 (adjusted *R*^2^ = −0.004).

However, when restrained eating was included as a covariate, an analysis of covariance (ANCOVA), which also included cue and awareness, and all interactions of cue, awareness, and restrained eating, with the percentage weight change as the dependent variable, revealed a three-way interaction of cue, awareness, and restrained eating, *F*(1,99) = 6.85, *p* = 0.010, η^2^_*p*_ = 0.07; η^2^ = $\frac{S\,S_{E\,f\,f\,e\,c\,t}}{S\,S_{C\,o\,r\,r\,e\,c\,t\,e\,d\,T\,o\,t\,a\,l}}$ = 0.06 (adjusted *R*^2^ = 0.061).

### Interaction of Cue and Awareness at High Values of Restrained Eating

Analyzing the interaction of cue and awareness at values of restrained eating with a moderation analysis, using the PROCESS macro for SPSS v3.4 (model 3 was used; Hayes, 2018), revealed the regions where cue and awareness interacted significantly (restrained eating values greater than 4.49 and less than 2.05, see Table 1; model summary: *R*^2^ = 0.123, *p* = 0.065).

**TABLE 1:** Conditional interaction of cue and awareness at values of restrained eating.

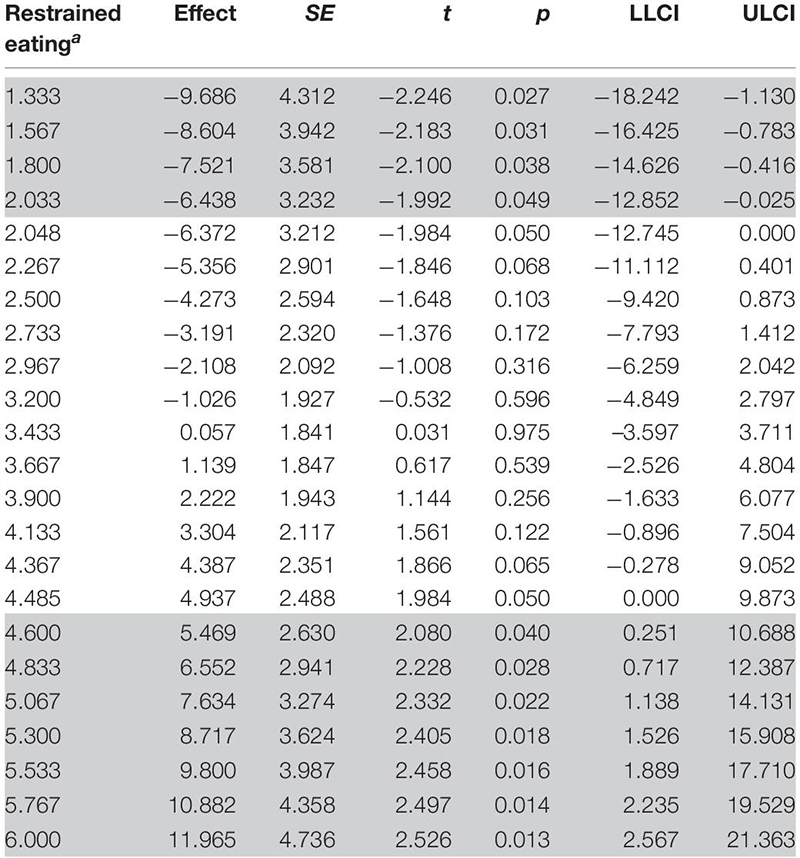

*^*a*^7-point Likert scale (1 = “*I do not agree at all”* to 7 = “*I entirely agree”*).*

*The shaded gray areas indicate the regions where cue and awareness interacted significantly.*

*Moderator value defining upper Johnson-Neyman significance region: 4.485, 25.23% of the values above (*n* = 27); moderator value defining lower Johnson-Neyman significance region: 2.048, 8.41% of the values below (*n* = 9).*

Focusing on the upper restrained eating region where cue and awareness interacted significantly—that is, on “highly restrained eaters” with restrained eating values greater than 4.49 (*n* = 27, 25.23% of the observations)—an ANOVA with cue and awareness as independent variables revealed a significant interaction of cue and awareness on participants’ percentage weight change, *F*(1,23) = 5.19, *p* = 0.032, η^2^_*p*_ = 0.18 (adjusted *R*^2^ = 0.146). Subsequent custom hypothesis tests (contrasts) revealed that, compared with the highly restrained eaters (i.e., with a restrained eating value >4.49) in the control condition *Rothko not aware*, to whom the visually neutral cue was not presented as a dieting facilitator (*M* = 0.86%, *SD* = 2.87), the highly restrained eaters in the experimental condition *Rothko aware*, who applied the visually neutral cue but were told that it was a dieting facilitator, lost weight (*M* = −7.72%, *SD* = 2.47), *p* = 0.012. Highly restrained eaters who applied the visually dieting-related Giacometti cue and were either not told, in the experimental condition *Giacometti not aware* (*M* = −3.06%, *SD* = 5.33), *p* = 0.142, or told that it was a dieting facilitator, in the experimental condition *Giacometti aware* (*M* = −2.63%, *SD* = 6.13), *p* = 0.199, did not significantly lose weight during the study compared with the highly restrained eaters in the control condition. Further, contrasts did not reveal any other statistically significant weight change differences among the participants of the experimental conditions.

Focusing on the lower restrained eating region where cue and awareness interacted significantly (restrained eating values <2.05, *n* = 9, 8.41% of the observations), an ANOVA did not indicate a significant weight change difference.

**FIGURE 1:**
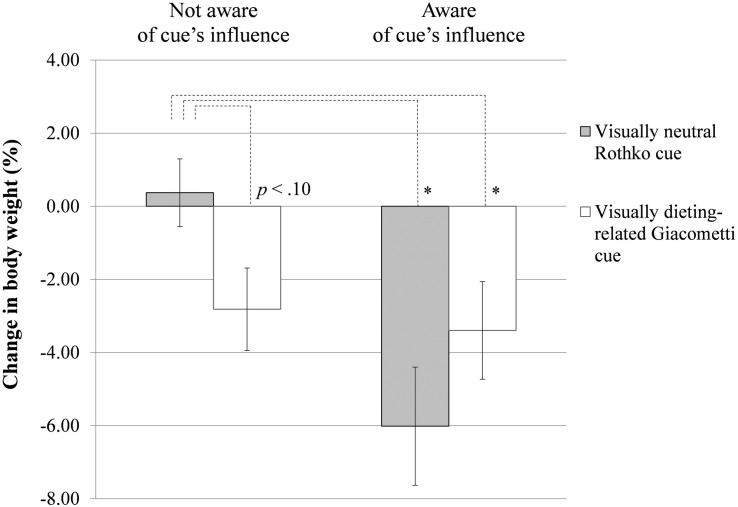
Mean change in body weight (in %) for restrained eaters derived from the median split, with a restrained eating value >3.50. Error bars represent standard errors. ^∗^*p* < 0.05.

To enlarge the limited sample size of the highly restrained eaters (*n* = 27) which resulted from the moderation analysis, the ANOVA and the subsequent custom hypothesis tests (contrasts) were repeated for the restrained eaters derived from the median split of restrained eating (*Mdn* = 3.50). The median split allowed us to take a closer look at a larger number of restrained eaters (*n* = 49). The ANOVA for the restrained eaters (restrained eating values >3.50) with cue and awareness as the independent variables revealed a significant interaction of cue and awareness on percentage weight change, *F*(1,45) = 5.17, *p* = 0.028, η^2^_*p*_ = 0.10 (adjusted *R*^2^ = 0.154). The custom hypothesis tests (contrasts) revealed that, compared with the restrained eaters in the control condition *Rothko not aware*, who applied the visually neutral cue and were not told that it was a dieting facilitator (*M* = 0.37%, *SD* = 3.47), restrained eaters in the experimental condition *Rothko aware*, who applied the visually neutral cue but were told that it was a dieting facilitator (*M* = −6.02%, *SD* = 4.28), lost weight, *p* = 0.002. Restrained eaters in the experimental condition *Giacometti aware*, who applied the visually dieting-related cue and were told that it was a dieting facilitator (*M* = −3.40%, *SD* = 4.99), *p* = 0.024, also lost weight compared with the restrained eaters in the control condition. Restrained eaters in the experimental condition *Giacometti not aware*, who applied the visually dieting-related cue and were not told that it was a dieting facilitator (*M* = −2.82%, *SD* = 4.22), tended to lose weight compared with the restrained eaters in the control condition, *p* = 0.055 (effect at *p* < 0.10); see Figure 1. Further, simple contrasts did not reveal any other statistically significant weight change differences between the participants of the experimental conditions.

The ANOVA for participants low in restrained eating (restrained eating values ≤3.50, *n* = 58) indicated an interaction of cue and awareness at the level *p* < 0.10, *F*(1,54) = 3.18, *p* = 0.080, η^2^_*p*_ = 0.06 (adjusted *R*^2^ = 0.048).

## Discussion

The present study examined (1) whether cues in the environment related to dieting, that is, environmental dieting cues, can be applied effectively over a longer period of time to lose weight, and (2) whether individuals can apply environmental dieting cues in a self-directed and deliberate manner, that is, when they are aware of being influenced by the environmental cue. Previous research has suggested possible contrast effects (i.e., diminished or reversed effects) when individuals are aware of being influenced (see Wegener and Petty, 1995; Zhu et al., 2015), and evidence on environmental dieting cues which are applied over a longer period of time is scant (Buckland et al., 2018). In the present study, dieters applied environmental dieting cues over a period of 6 months.

The results revealed a three-way interaction of the variables cue, awareness, and restrained eating with regard to participants’ percentage changes in body weight, as determined from weighing the participants at the briefing and the debriefing of the study. Highly restrained eaters, derived from the restrained eating region where cue and awareness interacted significantly, gained—on a descriptive level—some weight in the control condition *Rothko not aware*, in which participants applied a visually neutral cue (a picture of a Rothko painting) that was not presented to them as being a dieting facilitator. Compared with these highly restrained eaters in the control condition, highly restrained eaters in the experimental condition *Rothko aware*, in which participants applied the visually neutral cue that was presented to them as being a dieting facilitator, significantly lost weight. The same analyses conducted with a larger sample size, with the restrained eaters derived from the median split of restrained eating, revealed that the restrained eaters in the experimental awareness conditions, *Rothko aware* and *Giacometti aware*, significantly lost weight compared with the restrained eaters in the control condition *Rothko not aware*. Further, the analyses revealed the trend that the restrained eaters in the experimental condition *Giacometti not aware* with the visually dieting-related cue that was not presented to them as being a dieting facilitator tended to lose weight (*p* < 0.10) compared with the restrained eaters in the control condition.

In summary, the present study provides an initial indication that individuals with a strong chronic dieting goal, the restrained eaters (Herman and Mack, 1975), may be able to deliberately apply dieting-related environmental cues (i.e., to apply environmental cues which are presented to them as dieting facilitators) for themselves to facilitate losing weight over a longer period of time. Over the study period of 6 months, environmental dieting cues seemed to be most effective when the restrained eaters were aware that the cues supported weight loss. There were no indications of contrast effects. The results are consistent with previous evidence that highly valued or chronic goals facilitate the effects of environmental dieting cues (Stämpfli et al., 2017; Buckland et al., 2018). Further testing of the interaction of cue and awareness, over a longer period of time and with a larger sample size, is definitely required however.

### The Role of Awareness in Environmental Cues’ Effects and Limitations

Restrained eaters in the present study deliberately applied cues that were presented to them as being dieting facilitators with some success, and there were no indications of contrast effects. A plausible explanation is that contrast effects may more likely occur if individuals are aware of influences that are incongruent with their goals. In the present study, in which all the participants wanted to lose weight, it can be assumed that the environmental cues that were presented as dieting facilitators were perceived as goal-congruent.

The following evidence supports the assumption that contrast effects can occur for goal-incongruent influences but do not occur for goal-congruent influences of which individuals are aware. When individuals actually trained for a task, they performed better when they were told that the training had a positive influence than when they were told that the training had a negative influence (Nicolao et al., 2016). This suggests that individuals could counteract the actual positive influence from the training when they believed that the training had a negative influence. Importantly, this effect only occurred when the training actually prepared individuals for the subsequent task. This rules out a mere placebo effect (Nicolao et al., 2016)—that is, an effect which cannot be attributed to an actual treatment but to individuals’ belief of being treated (Fontaine et al., 2016).

Previous research on dieting has indicated that placebo effects can affect weight loss (Panayotov, 2019; see Fontaine et al., 2016). For instance, hotel room attendants who were told that their work-related physical activity exceeded the recommended amount lost weight within 4 weeks, independent of their actual physical activity levels (Crum and Langer, 2007). In the present study, the weight loss effect of the visually neutral cue on participants who believed it to be a dieting facilitator could be considered a placebo effect. However, as we assume that the environmental dieting cues used in this study influenced behavior by activating the dieting goal (see Papies, 2016b), individuals could be seen as treated if the cues actually activated their dieting goal, also when the cue *per se* was visually neutral.

As a limitation of the present study, stronger demand effects (Sawyer, 1975) for participants in the awareness conditions, and especially in the condition *Rothko aware* in which participants applied a visually neutral cue and were told that it was a dieting facilitator, cannot be ruled out. In terms of limitations of the present study, it should also be noted that the participants differed in whether they applied the stickers and how often they had seen them, according to their self-reported information. Exposing study participants to a cue more reliably, such as via smartphone alerts, should improve future studies. Further, regarding limitations of the present study, it has to be considered that we did not control for other weight management practices—that would have exceeded the weight management practices imposed by the study’s design—in which participants engaged in during the study. Therefore, it is possible that there were supporting influences regarding losing weight, such as concurrent dieting support from a physician. Another limitation is that the study was not preregistered. As a last limitation that should be mentioned, the effects of intervention studies can diminish after the intervention ends (for example, Inauen et al., 2017). After the present study had ended, the weight development of the participants was not tracked.

### Goals That Facilitate Effects of Dieting Cues Over a Longer Period of Time

In the present study, we found that restrained eaters with their highly valued chronic dieting goal (Papies, 2016b; Weingarten et al., 2016; Polivy and Herman, 2017) could deliberately apply environmental dieting cues to facilitate the pursuit of their personal goal of losing weight. This is consistent with previous evidence showing that activated goals have a stronger effect on behavior when the goals are highly valued by the individuals (Weingarten et al., 2016). Interestingly, although all of our participants could be assumed to have had highly valued dieting goals, given that they chose to participate in a 6-month dieting study, the dieting cues only had an effect on the restrained eaters. As restrained eating was defined to be chronic dieting (Polivy and Herman, 2017), this may suggest that an environmental dieting cue that is implemented over a longer period of time must activate a persistent or chronic goal in order to elicit longer-term effects. Thus, previous evidence (Buckland et al., 2018) and the results of the present study suggest that, while environmental dieting cues can generally have immediate effects on individuals who highly value their dieting goal—that is, also on individuals who may value a goal only shorter-termed such as dieters—effects over a longer period of time require a highly valued persistent dieting goal.

In addition to persisting goals, perceived goal attainability could have facilitated the effects of the environmental cues in the present study that were presented to the participants as dieting facilitators. Accepted as such, the cues could have allowed participants’ goal of losing weight to appear more attainable. Previous research has indicated that perceived goal attainability plays a role in the effectiveness of environmental dieting cues (Klesse et al., 2012). Perceived goal attainability could be decisive for enabling the positive affect related to a goal or the related dieting cue to drive goal pursuit (Anschutz et al., 2008a; Custers and Aarts, 2010). A conceivable way to enhance perceived goal attainability could be social support, for instance, via smartphone chat messages (Inauen et al., 2017).

### Implications for Future Research

There is little evidence about the effects of environmental dieting cues over longer periods of time (Papies, 2016b; Buckland et al., 2018). Indeed, some health intervention studies have examined the effects of environmental cues on food choices, sales, or consumption in places such as restaurants and cafeterias over time spans of up to several months (see Allan et al., 2017; Cadario and Chandon, 2018; see Carter et al., 2018). Such public health intervention studies are of significant practical value. However, they do not allow researchers to examine the effect of repeatedly presented environmental cues on particular individuals—with the individuals’ characteristics such as restrained eating which can be decisive for the cues’ effects. This is because, in public eating settings, the clientele does not remain constant. While it may remain relatively constant in on-site eating settings (e.g., in universities or worksite cafeterias), this will not be the case in off-site eating settings such as restaurants, cafes, or cinemas. Thus, mainly short-term effects of environmental cues on different individuals are captured when health intervention studies are evaluated. In line with this, a meta-analysis of public health intervention studies in on-site eating settings, off-site eating settings, and grocery stores found that the effects of healthy eating interventions were largely independent of study duration. Increasing the study duration from 1 to 15 weeks reduced the effect size by only 13% (Cadario and Chandon, 2018).

Thus, further studies are needed to examine the effects of repeatedly presented environmental dieting cues on specific individuals—ideally, with individuals who apply the cues deliberately for themselves. Such studies should address various temporal matters (Scholz, 2019). For example, they could shed light on the aspect of habituation. In general, to apply a distinct cue repeatedly could lead individuals to habituate to the cue, which would involve a decrease in their behavioral response (Rankin et al., 2009; Silvestrini and Gendolla, 2011; Papies, 2016b). Importantly, future studies should have large enough sample sizes. Further, as in the present study it was not controlled for all weight management practices that participants engaged in during the study, which could have supported losing weight, studies which control for more potentially intervening influences are needed to confirm the present study’s findings. In formal terms, future studies should also have preregistered research protocols that indicate, among other factors, the research hypotheses and criteria for data exclusions.

### Making the Healthy Choice the Easy Choice

Today’s obesity epidemic with its increased number of obese adults (World Health Organization [WHO], 2020b) indicates the relevance of the present study. In our obesogenic environment (Hill et al., 2003; Papies et al., 2014), to deliberately use environmental dieting cues in tempting situations—for example, at the fridge—to activate our dieting or healthy eating goals could help. An advantage of this approach is that it can be pursued with minimal effort. In contrast, interventions which attempt to modify (instead of activating elements of) the existing cognitive structures that underlie individuals’ health-related behavior, such as making specific plans for how to behave in response to tempting situations, mostly involve a form of training which is normally time-consuming (Haasova et al., 2016; Papies, 2016b).

However, the effects of environmental dieting cues over a longer period of time may depend not only on the presence of highly valued goals which persist over time, as suggested by the present research, but are dependent on individuals’ possibility to pursue their health goals (Papies, 2016b). This possibility is facilitated by environments in which healthy food options are available, accessible, and salient (Papies, 2016b, 2017; see also Kwasnicka et al., 2019). This is in line with *nudging* (Thaler and Sunstein, 2008; see also Hansen and Jespersen, 2013). Nudging is used as an “umbrella term” for influences of the environment on individuals’ behavior—of which the influenced individuals are usually not aware (Chartrand, 2005)—and has been more specifically defined as enhancing the accessibility or salience of behavioral options to change behavior (Papies, 2017). Placing healthy products near the cash register (Kroese et al., 2016) is an example of a nudging intervention, as is listing healthy food options first on menus and/or highlighting them as healthy (Policastro et al., 2017; as an existing example see the Nutri-Score, Giner and Brooks, 2019). According to the World Health Organization [WHO] (2018), overweight and obesity can be reduced by supportive environments that make the choice of healthier foods the easiest choice (“the choice that is the most accessible, available, and affordable”). Activating dieting goals using environmental cues, ideally with individuals who apply the cues deliberately for themselves according to the results of the present study, in combination with a supportive environment in terms of improved availability, accessibility, salience, and affordability of healthy food options, could thus represent a promising strategy to address the problem of obesity. With regard to the limitations of the present study, however, the present study should be seen as a starting point for future improved long-term studies, which, for example, could have a larger sample size and control for more potentially intervening influences.

## Data Availability Statement

All relevant data is contained within the article: The original contributions presented in the study are included in the article or the Supplementary Material. Further inquiries can be directed to the corresponding author.

## Ethics Statement

The study involving human participants was reviewed and approved by the ethics commission of the canton of Bern (KEK No. Z047/13). The participants provided their written informed consent to participate in this study.

## Author Contributions

All authors contributed to the study design and approved the final manuscript. AS analyzed the data and wrote the manuscript. SS, TB, and CM contributed their input.

## Conflict of Interest

The authors declare that the research was conducted in the absence of any commercial or financial relationships that could be construed as a potential conflict of interest.
