# Supplementary figures and images for: A Dieting Facilitator on the Fridge Door: Can Dieters Deliberately Apply Environmental Dieting Cues to Lose Weight?
Source: Front Psychol. 2020 Dec 21;11:582369. doi: 10.3389/fpsyg.2020.582369 (PMC7779523; doi:10.3389/fpsyg.2020.582369)

## Supplementary Figure

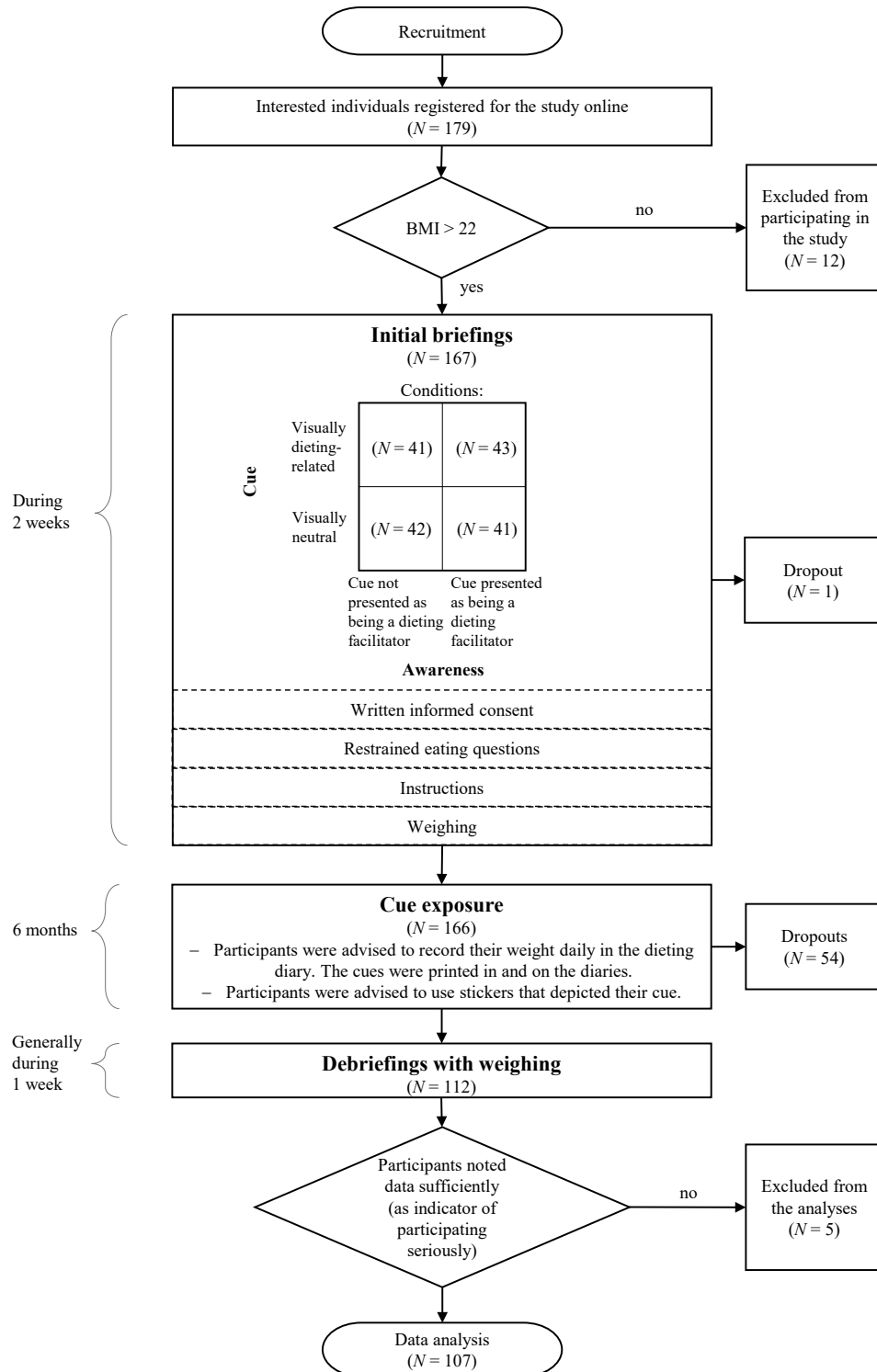

**Supplementary Figure 1.** Flowchart: Procedure.

Supplement: Supplementary file 2 [file Image_1.pdf]
